# Collective efficacy and the built environment*

**DOI:** 10.1111/1745-9125.12304

**Published:** 2022-02-15

**Authors:** Charles C. Lanfear

**Affiliations:** ^1^ University of Oxford

**Keywords:** built environment, collective efficacy, routine activities, situational opportunity

## Abstract

Collective efficacy is a prominent explanation for neighborhood crime concentrations. Just as crime is concentrated in particular neighborhoods, within‐neighborhoods crime is concentrated in particular criminogenic locations. Research suggests criminogenic locations are determined by features of the built environment. This study links collective efficacy with situational opportunity to propose that collective efficacy facilitates the removal of criminogenic features of the built environment. I test this by examining associations 1) between past collective efficacy and present criminogenic features of the built environment, as well as 2) between those built environment features and crime, net of present collective efficacy. These are modeled using piecewise structural equations with generalized linear mixed‐effect regressions on data from 1,641 blocks in 343 Chicago neighborhoods. Four types of police‐reported crime are modeled using eight block‐level built environment features in the 2003 Chicago Community Area Health Study (CCAHS; N = 3,074) and neighborhood collective efficacy from the CCAHS and the 1995 Project in Human Development in Chicago Neighborhoods (PHDCN) Community Survey (N = 7,672). Findings suggest neighborhoods with high collective efficacy maintain low rates of crime in part by limiting criminogenic built environment features, in particular, abandoned buildings. This crime control pathway is important because changes to the built environment are long lasting and reduce the need for future interventions against crime.

## 1 INTRODUCTION

Crime is highly concentrated in particular urban neighborhoods (Shaw & McKay, 1942/1969). Variation in collective efficacy—a problem‐solving capacity of neighborhoods—is a prominent explanation for neighborhood differences in crime (Sampson, 2012; Sampson et al., 1997). Informal social control, such as resident monitoring and interventions against crime and disorder, is assumed to be the primary mechanism by which collective efficacy reduces neighborhood crime. Importantly, collective efficacy measures the perceived capacity for such social control actions, rather than realized actions, which occur only when an offense is attempted (Sampson, 2012, pp. 156−160). Even when interventions are not observed, the perceived certainty of intervention exerts a deterrent effect on crime in neighborhoods with high collective efficacy. Like the classic social disorganization construct from which it is intellectually descended, collective efficacy mediates much of the relationship between neighborhood sociodemographic structure and crime (Sampson et al., 1997).

Just as crime is concentrated in particular neighborhoods, within‐neighborhoods crime is concentrated in particular locations—hot spots (Sherman et al., 1989; Weisburd et al., 2012). The locations of hot spots are mainly determined by the presence of features of the built environment that are criminogenic in the sense that they provide opportunities for crime (Brantingham & Brantingham, 1991; St. Jean, 2007; Wilcox & Cullen, 2018). The criminological literature is rich with examples of these features, such as abandoned buildings (Spelman, 1993), venues for alcohol sales (Roncek & Maier, 1991), commercial properties and mixed land use (Taylor et al., 1995), and recreation facilities (Weisburd et al., 2009).

Residents, rightly or not, associate features of the environment such as abandoned buildings with crime and disorder and consequently view them as problematic (Innes, 2004). As a problem‐solving capacity of neighborhoods, collective efficacy may facilitate actions to remediate, remove, or prevent the development of these features. In contrast to the more commonly studied informal control interventions to control unwanted behavior, these actions are interventions to control contexts perceived to precipitate unwanted behavior. If collective efficacy promotes the control of criminogenic features of the built environment, then the concentration of these features should partially explain the effect of collective efficacy on crime.

Accordingly, this work examines how collective efficacy is related to the distribution of criminogenic features of the built environment, as well as the contribution of those features to rates of crime. I base this in a framework that integrates collective efficacy with situational opportunity. This framework flows from past research uniting theories of neighborhood social context and criminal opportunity (e.g., Bursik & Grasmick, 1993; Miethe & McDowall, 1993; Wilcox & Tillyer, 2017; Wilcox et al., 2003). When considering the built environment, this literature typically focuses on cross‐level moderation: how effects of the built environment on crime differ by neighborhood context. The present work differs by examining cross‐level mediation: how the built environment itself is affected by neighborhood context (past collective efficacy) and how this in turn affects crime rates. I test hypotheses from this framework with a multilevel longitudinal research design using data from Chicago on block‐level built environment features and neighborhood‐level collective efficacy. My findings suggest neighborhoods with high collective efficacy maintain low rates of crime in part by limiting the presence of built environment features that provide criminal opportunities. Although not the focus of the present research, appendix 3 in the online supporting information also includes an examination of cross‐level moderation, for which I find modest evidence.^1^

### 1.1 The built environment and crime

The built environment has long been recognized as a one of the most important predictors of crime (e.g., Jacobs, 1961; Jeffery, 1977; Newman, 1978). Although the built environment influences many forms of crime, the present research focuses specifically on crimes defined as direct‐contact predatory violations—acts in which an offender intentionally directly and physically takes or damages another individual or their property (Cohen & Felson, 1979). By structuring the routine activities of people, the built environment influences the requisite components of these predatory criminal acts: the convergence in time and space of motivated offenders, suitable targets, and the absence of capable guardians (Cohen & Felson, 1979; see also Brantingham & Brantingham, 1991).

Many features of the built environment are potentially criminogenic, but importantly, they are not purely criminogenic: A park may provide recreation to families, provide concealment for criminal activities, or both, perhaps depending on the time of day. In this way, potentially criminogenic features of the built environment also serve noncriminal purposes, and thus, they are not perceived solely as problematic. Simply removing all features that might facilitate crime is not a valid solution because they are necessary for the routine activities of people. Crime tends to be higher in the presence of most nonresidential features of the built environment simply because more people make use of those spaces (Brantingham & Brantingham, 1991; Wilcox & Eck, 2011). Crime cannot exist in a vacuum but neither can people. Control of crime facilitated by the built environment exists in tension with the legitimate uses of space. Criminogenic features perceived to offer little benefit to residents—such as abandoned buildings and vacant lots—are thus likely to be subject to stronger removal efforts.

Situational opportunity theories of crime—including routine activities theory—posit that different contexts generate opportunities for different types of crime (Cohen & Felson, 1979; Wilcox & Cullen, 2018). For instance, an unattended home provides an opportunity for burglary, but not homicide or robbery, because no one is home. Similarly, criminogenic effects of built environment features are specific to particular criminal opportunities. For example, vacant lots and abandoned buildings facilitate homicide and gun violence by acting as illicit firearm storage (MacDonald et al., 2019); liquor stores and bars precipitate assaults and provide vulnerable targets for robberies (Pridemore & Grubesic, 2012; Wheeler, 2019); commercial or mixed land uses—as well as parking lots—impede social control and provide targets for robbery and property crime (Browning et al., 2010; Taylor et al., 1995; Wo, 2019; see also Taylor, 1988); and recreation facilities present a range of opportunities by attracting visitors and promoting unstructured socializing of youth (Boivin & Felson, 2018; Osgood et al., 1996; Weisburd et al., 2009). Some features may, like many nonresidential properties, promote a wide range of crimes by increasing the number of people present (Cohen & Felson, 1979; Tucker et al., 2021; Wilcox & Eck, 2011), or like the layout of streets, by impeding (or facilitating) mobility (Greenberg et al., 1982; Johnson & Bowers, 2010). The effects of built environment features on crime often extend beyond the feature itself (Groff & Lockwood, 2014; Ratcliffe, 2012) in part as a result of the effects on mobility (Brantingham & Brantingham, 1991).

These are, of course, only a selection of relevant examples. The literature on opportunity and the built environment is voluminous (for overviews, see Taylor & Harrell, 1996; Wilcox & Cullen, 2018; Wuschke & Kinney, 2018). An outstanding question, and the focus of this work, is the degree to which collective efficacy influences the distribution of these criminogenic features of the built environment.

### 1.2 Control of the built environment

The built environment is shaped by the actions of local government in conjunction with developers and property owners (Logan & Molotch, 2007). Neighborhood residents may work collectively to control crime by removing, remediating, and preventing development of features that are perceived to present criminal opportunities. In this way, residents can use their economic, social, and political capital to influence external institutions and constrain criminal opportunities. Although prominent research in community social control acknowledges the role of institutional linkages in shaping neighborhood conditions (e.g., Bursik & Grasmick, 1993; Sampson, 2012; Velez, 2001), the role of the built environment has not been specified within a general collective efficacy and situational opportunity framework.

Action to alter the built environment is likely dependent on connections with external institutions and actors such as developers and policy makers. Even where the actors responsible for a given criminogenic feature are themselves neighborhood residents—such as the owner of a problematic bar—external institutions with formal authority to sanction owners provide a point of leverage for collective action (Carr, 2005, pp. 121−123). The social disorganization tradition—from which collective efficacy emerged—has long recognized the importance of the community's relationship to external actors. For example, in the systemic model of social disorganization, disorganized neighborhoods are characterized by an absence of connections to and influence over external institutions, such as city government (Bursik & Grasmick, 1993). Collective efficacy is thought to predict political mobilization to influence external institutions (Sampson, 2012, pp. 152−153), and one of the most commonly used indicators of collective efficacy is expectations that residents would organize to defend a fire station (or library) from closure (Sampson et al., 1997). This describes collective political action to influence local government to maintain an existing beneficial built environment feature.

Informal social control and control of the built environment are parallel forms of problem‐solving that may emerge from the same latent capacity for action. Collective efficacy activates as informal social control when residents believe acts like verbal sanctioning can address problem behavior. When residents perceive features of the built environment as the source of criminal behavior or other threats, they sometimes engage in direct clean‐up and remediation efforts that are analogous to the direct interventions associated with informal social control (Kelling & Coles, 1996). In other cases, however, collective efficacy activates as political action to influence external actors with authority to address the problem. For example, Einstein et al. (2020) observed residents of a wealthy neighborhood fighting to prevent construction of a low‐income housing development by lodging complaints in zoning board meetings, filing lawsuits, and petitioning officials. The residents described this as action on behalf of their community to protect it from crime, neighborhood change, and harm to property values. This represents an activation of collective efficacy as political action. Other examples of actions directed at external institutions include protests (Rabrenovic, 1996), invocations of regulatory agencies (Carr, 2005), and collaborative policy development (Donnelly & Kimble, 1997).

An important characteristic of collective efficacy is that it represents a capacity for social control actions rather than the frequency of those actions. Collective efficacy is assumed to reduce crime not only by promoting interventions but also through a deterrent mechanism (Sampson, 2012, pp. 159−160): Individuals are deterred from attempting offenses in highly efficacious neighborhoods because they perceive interventions by residents to be likely. A similar mechanism may operate with regard to the built environment. Efficacious neighborhoods can turn attempts at development into extended, costly battles (Einstein et al., 2020). If developers and city officials anticipate a particular neighborhood will be highly resistant, they may be unlikely to consider that neighborhood for their development. When a development is undesirable to residents but its location is flexible—a jail for example—disadvantaged neighborhoods become the default locations of first consideration (Logan & Molotch, 2007, p. 113). In this way, absent any observed political action, collective efficacy can still prevent the emergence of features residents perceive as undesirable, some of which are likely criminogenic (but some of which are not, e.g., Bursik, 1989).

Unlike informal and formal control that operate immediately to inhibit crime, the slow pace of change in the built environment makes it a subtle and enduring method of crime control. Neighborhoods with high collective efficacy in the past may exhibit low crime in the present because they prevented the emergence of criminogenic features. Given changes to the built environment are slow and cumulative, the built environment should be a mediator with regard to crime only for past collective efficacy. Furthermore, if past collective efficacy impacts the built environment, which in turn impacts present collective efficacy, then change in the built environment is a mechanism by which collective efficacy is propagated over time and may serve as a point of intervention to bolster collective efficacy. This may occur if some features foster social ties and cohesion—building blocks of collective efficacy—by increasing interaction between residents (Browning, Calder, Soller, et al., 2017; see also Small & Adler, 2019). The converse may also occur if unwanted features of the built environment reduce residents’ use of public spaces and attachment to the neighborhood (Branas et al., 2018; Taylor et al., 1985).

The collective action of residents is not the only means by which features of the built environment change. The built environment of neighborhoods also responds to the political economy of the city and region. Rising (or declining) property values, or the anticipation of rising (or declining) property values, leads to changes in behavior by external actors like developers. External actors seeking to maximize the value of their property holdings for investment purposes often operate at odds with residents focused on maximizing the livability of their homes and neighborhoods (Logan & Molotch, 2007). Neighborhoods with organized, wealthy, or politically influential residents—those more likely to be collectively efficacious—more easily resist changes that compromise their perceived quality of life (Logan & Molotch, 2007). Einstein et al.’s (2020) neighborhood resistance against affordable housing provides an example. Disadvantaged neighborhoods—those less likely to be collectively efficacious—are more vulnerable to actions by outside actors looking to maximize their investments at the cost of resident quality of life. This includes nonresident owners of dilapidated apartments or poorly regulated bars that extract money from neighborhoods with little concern for residents (Desmond, 2016; Eck & Madensen, 2018). As a result, even though the built environment of neighborhoods is subject to powerful outside forces, the ability of those outside forces to enact their will is in large part dependent on neighborhood socioeconomic structure and capacity for collective action.

## 2 APPROACH

Based on this theoretical framework, I test the following hypotheses:

Hypothesis 1
The features of the built environment facilitate crime by promoting convergences of potential offenders and suitable targets in the absence of capable guardians. Specifically:
  a) The features characterized by valuable or unguarded property or people carrying valuable property, including commercial destinations, mixed land use, and parking lots, will promote property crime and robbery.
  b) The features that reduce inhibitions, precipitate conflicts, or conceal weapons and illicit market transactions, such as abandoned buildings, bars, and vacant lots, will promote violence.
  c) Nonresidential features in general—and recreation facilities in particular—may promote crime by increasing the number of people present at any given time.

Hypothesis 2
Past collective efficacy reduces the presence of criminogenic features of the built environment.

Hypothesis 3
Criminogenic features of the built environment reduce collective efficacy.

**FIGURE 1:**
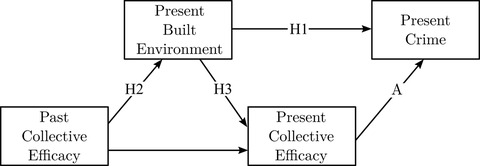
Theoretical model of collective efficacy, the built environment, and crime *Note*: Tested hypotheses represented as paths H1, H2, and H3

Figure 1 depicts these hypotheses graphically. For simplicity, exogenous controls like sociodemographic structure are omitted. A basic assumption of these hypotheses is that collective efficacy is negatively related to crime in the short term (path A). This is generally supported in the literature (Lanfear et al., 2020). Although not a focus of the present study, this analysis serves as a replication of this past research. If hypotheses 1 and 2 (or 1 and 3) are both supported (paths H1 and H2 are nonzero), then criminogenic built environment features are confounders that, when omitted, exaggerate the contemporaneous effect of collective efficacy on crime. It is possible the direct effects of collective efficacy on crime (A) may be greatly, or even fully, attenuated once adjusting for features of the built environment. Even if this were the case, if hypothesis 2 is supported, it would suggest collective efficacy is still relevant to crime control, through the mechanism of control of the built environment rather than the assumed primary mechanism of informal social control.

Although this study proposes a causal relationship between collective efficacy and crime via the built environment (hypotheses 1 and 2 combined), this is difficult to test. At the very least, there are three challenges: 1) (sequential) ignorability, 2) reciprocality, and 3) task specificity of collective efficacy. I summarize these briefly here.

First, collective efficacy and the built environment are not randomly assigned characteristics of neighborhoods. Estimating causal effects requires assuming that treatment assignment (e.g., the presence of abandoned buildings) is approximately random—that is, the assignment process is ignorable (Rosenbaum & Rubin, 1983). It is reasonable to assume ignorability will hold in experiments with randomized treatments. Ignorability is unlikely to hold in observational research where treatments are assigned to units through natural processes, as is the case for collective efficacy and built environment features. In this context, causal effects may be estimated under the weaker assumption of conditional ignorability—that assignment is approximately random conditional on pretreatment covariates predicting both the treatment and the outcome of interest (Robins & Greenland, 1992; Rosenbaum & Rubin, 1983).

Urban development—the assignment process of built environment features—is the product of an interaction between the political economy of metropolitan areas and the current and historical structural characteristics of neighborhoods, including social and political capital of residents (e.g., collective efficacy) (Dreier et al., 2014; Logan & Molotch, 2007). Collective efficacy is the product of a neighborhood's history, sociodemographic structure, and relative position compared with other neighborhoods in the city (Sampson, 2012; Sampson et al., 1997). The analyses that follow adjust for measures of these social‐structural factors that predict assignment of built environment features and collective efficacy. These covariates will also absorb some effects of omitted variables that are highly correlated with them or mediate their effects—such as public infrastructure, zoning, and present or anticipated land values (Logan & Molotch, 2007). Although conditional ignorability—and thus causality—is not firmly established, the structural models below explain nontrivial variation in the built environment features and collective efficacy, and the estimated direct effects are robust to various specification tests.

Identifying causal mediation—the effect of past collective efficacy on present crime via features of the built environment—requires the additional assumption of sequential ignorability (Imai et al., 2010): Assignment of the mediator must be ignorable conditional on both treatment and included pretreatment confounders. This assumption is strong and generally untestable even when treatment is randomized (Sobel, 2008). Complicating this is the presence of multiple correlated mediators (i.e., the built environment features) that complicate identification of indirect pathways even if sequential ignorability holds (VanderWeele, 2015). As a result, the conditional direct effects—collective efficacy on the built environment—may be tested more convincingly than the indirect effects—collective efficacy on crime via the built environment. The following analyses focus on conditional direct effects for this reason—in particular the effect of past collective efficacy on the built environment, as causal effects of built environment features on crime are well established (Kondo et al., 2018).

Second, it is likely that some built environment features foster collective efficacy, creating a positive feedback loop over time (hypothesis 3). For example, successful removal of criminogenic features may embolden residents to undertake more efforts in the future. Some features of the built environment may also increase social interaction that in turn strengthens collective efficacy (Browning, Calder, Soller, et al., 2017; Small & Adler, 2019). Endogeneity of this sort will bias estimates upward. Provided repeated observations of neighborhoods, this may be addressed with longitudinal models. In the present case, only neighborhood collective efficacy is measured at two time points, and not block‐level crime or the built environment, preventing use of a conventional panel model. I address this by predicting built environment features using past collective efficacy, and predicting present collective efficacy using present built environment features. This makes the assumption that residents adapt to changes in the physical environment more quickly than collective efficacy influences the built environment.

Third, and finally, collective efficacy is task specific (Sampson et al., 1997). Measures of collective efficacy are designed to capture informal social control capacity. This study, however, is concerned with residents’ capacity to control the built environment, which likely occurs primarily via political action. I expect these factors will be strongly correlated in part because one common indicator of collective efficacy is expectations residents would intervene to protect a fire station or library—actions to control the built environment. Nonetheless, I expect the standard measure of collective efficacy to be more strongly associated with crime directly—implicitly via informal social control—than indirectly via the built environment. This may attenuate the estimated effect of collective efficacy on the built environment.

Even if frequently unarticulated, all observational research on community crime and social control rests on assumptions like those described above, in particular, regarding ignorability and reciprocality (Lanfear et al., 2020; see also Taylor, 2015, ch. 9). When studying community‐level processes that operate over years, these also cannot easily be relaxed, such as via experiments (Nagin & Sampson, 2019). Making these assumptions explicit is necessary to enable readers to fairly assess research designs and develop new designs that address these assumptions.

## 3 DATA

This analysis uses data from the 2001 through 2003 Chicago Community Adult Health Study (CCAHS; House et al., 2011). The CCAHS was administered to a stratified, multistage sample of 3,105 adults living in Chicago. This survey is used to construct a measure of collective efficacy at the neighborhood cluster level—the primary stratification unit for the survey. These clusters were originally created for the 1995 Project in Human Development in Chicago Neighborhoods (PHDCN) to represent Chicago neighborhoods (Earls et al., 1999). Each cluster is a set of, on average, three geographically contiguous census tract. The median cluster is 0.50 square miles in area, and 90 percent of clusters are between 0.19 and 1.61 square miles.^2^ These clusters were constructed to maximize ecological validity using a combination of cluster analyses of census‐recorded sociodemographic characteristics to ensure internal homogeneity, natural boundaries from prominent geographical features (e.g. freeways), as well as local knowledge of Chicago neighborhoods (Sampson, 2012, pp. 78−80; Sampson et al., 1997, p. 919). Hereafter I use the term “neighborhood” to refer to these units and refer to measures from the 2001−2003 CCAHS as 2003 measures.

In line with past research, I measure collective efficacy as a combination of resident expectations their neighbors would intervene against different types of deviance—but also to protect a library or fire station threatened with defunding—and perceptions of cohesion and trust—such as shared values in the neighborhood (Sampson et al., 1997). As is common in this literature, my measure of collective efficacy is an empirical Bayes estimate derived from a multilevel measurement model that adjusts resident‐perceived collective efficacy for sociodemographic characteristics of respondents and corrects for measurement error (Sampson et al., 1997). This measurement model was estimated using 3,074 (99 percent) observations with sufficient data for subsequent measurement models. See appendix 1 in the online supporting information for indicators and neighborhood reliabilities.

Neighborhood sociodemographic structure is a primary determinant of crime rates, and collective efficacy mediates a portion of this relationship (Sampson et al., 1997). To properly specify models of collective efficacy and crime, I constructed measures of the neighborhood sociodemographic structure. Following past research in this area (e.g., Sampson et al., 1997), I generated a parsimonious set of measures using an alpha‐scoring oblique factor rotation of nine‐year 2000 census indicators from the Longitudinal Tract Data Base (LTDB; Logan et al., 2014). Despite being conducted in 2001−2003, the CCAHS data is identified to year 1990 census boundaries. The LTDB normalizes tract boundaries to ensure measures describe the same units over time. The indicators were chosen to match those used by Sampson et al. (1997) to operationalize 1990 neighborhood social‐structural characteristics, although one of these indicators (families on public assistance) was not available in the LTDB.^3^ Based on the factors each indicator loads on, I label the factors “disadvantage,” “stability,” and “Hispanic/immigrant” population. These factors are analogous to the classic structural antecedents of social disorganization and its modern derivatives (Bursik & Grasmick, 1993; Shaw & McKay, 1942/1969). See appendix 2 in the online supporting information for a list of indicators and their factor loadings.

**FIGURE 2:**
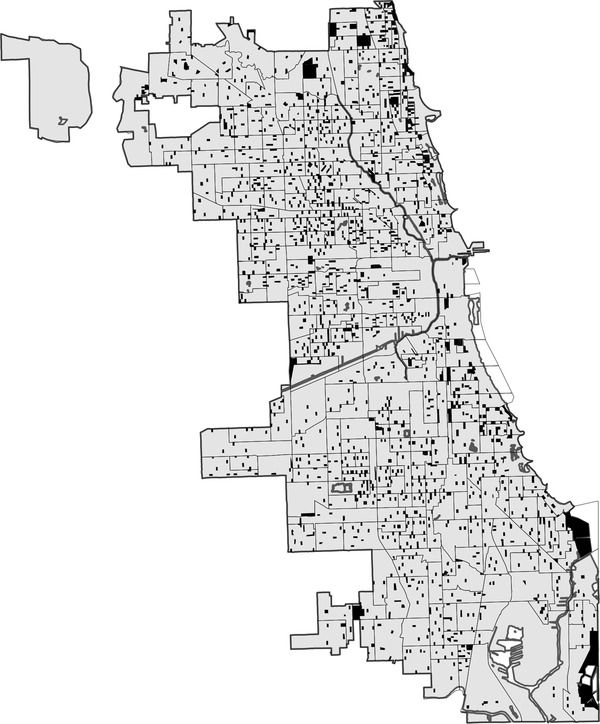
Map of census blocks sampled in 2001−2003 Chicago Community Adult Health Study *Notes*: Sampled blocks are filled black shapes. Neighborhood clusters are outlines

The CCAHS also provides systematic social observation (SSO) measures of a random sample of census blocks within each neighborhood cluster—the same blocks in which respondents resided. The SSO for the CCAHS was conducted by survey interviewers walking the perimeter of the sampled block twice and recording what they observed on each street segment and block face via a checklist. A block face is a single side of a street segment between the intersections that form corners of a block. Observers recorded data for block faces on the focal block, as well as block faces on adjacent blocks that face the focal block. A rectangular block thus has four street observations and eight block face observations. The indicators recorded cover a broad range of features describing health hazards, the built environment, and disorder (see House et al., 2011). The SSO for the CCAHS covers all 343 neighborhood clusters; however, only 1,641 of Chicago's approximately 20,000 census blocks are represented. Figure 2 depicts the sampled blocks and neighborhood clusters. From the SSO, I obtain measures of all built environment features: abandoned buildings, bars, commercial destinations, liquor stores, mixed land use, parking lots, recreation facilities, and vacant lots. All built environment measures are proportions of block faces or streets on and surrounding the census block that have that feature present.

It is important to note that these features are spread across a continuum of perceived desirability by residents. Abandoned buildings have no positive functions for any neighborhood residents, with the possible exception of property owners awaiting rising values to redevelop or sell. In contrast, most commercial destinations and recreation facilities provide valuable amenities to the neighborhood. The degree to which collective efficacy is associated with the presence of these features is likely governed by the perceived balance of positive and negative impacts they make to the neighborhood. That is, even if a built environment feature—like a local park—is associated with crime, residents may not advocate for the removal of the property if it provides use value that outweighs the cost of crime. In this way I diverge from studies that focus on criminogenic effects and control only of “unpopular places” (Wilcox & Eck, 2011). It may be the case that many features are comparably criminogenic, but removal efforts are concentrated on those features perceived as particularly undesirable or unambiguously problematic.

To analyze the impact of past collective efficacy on the present built environment, I construct a past collective efficacy measure from the 1995 PHDCN community survey (PHDCN‐CS; *N* = 8,782). I estimate this with a multilevel measurement model on the 7,672 (87 percent) observations with sufficient data for estimation. The PHDCN‐CS is similar to the CCAHS's community survey but produces more precise estimates of neighborhood social structures as a result of its larger sample size. The PHDCN SSO is not used for block‐level analyses here because it was conducted in only 80 neighborhood clusters, and block‐level identifiers are not available to link those blocks across surveys.

The neighborhood and block measures were linked to publicly available geocoded Chicago Police Department data from the three years after the CCAHS (2004−2006) to obtain block‐level counts of crime incidents (Chicago Police Department, 2020). A three‐year span was used because serious crimes are rare at the block level and using multiple years reduces the influence of idiosyncratic variation. I consider four forms of crime: 1) homicide and assaults featuring a gun, 2) robbery, 3) any violent crime (defined as homicide, robbery, and assaults with or without a gun), and 4) any property crime (defined as any burglary or theft). These forms of crime were chosen for two reasons. First, they are direct contact predatory violations likely to be particularly sensitive to different opportunities structured by the built environment. Second, accuracy of reporting tends to be higher for more serious crimes such as homicide and gun violence. Homicide and gun violence were pooled because homicide is rare at the block level, and separate models for each outcome exhibit similar relationships with the predictors, although with wide confidence intervals for homicide alone.

Block‐level measures of population density and adjacent street types were included to adjust for ambient population. Despite being collected in 2001−2003, CCAHS blocks were identified using 1990 census block boundaries to match the PHDCN. Consequently, 2000 census block populations were areal weighted to 1990 boundaries where those boundaries changed across decennial censuses. Areal weighting is a process in which values describing one geographic area are assigned to another in proportion to the area of their intersection, under the assumption the values of interest are distributed uniformly in space. These resulting population values were then divided by block area to arrive at a population density.^4^ This is a block‐level analog to the tract‐level normalization process for the LTDB used to construct neighborhood‐level measures. Lastly, a street grid shapefile from the City of Chicago was used to generate an ordinal Street Class control variable (City of Chicago, 2011). Blocks were assigned the value of their highest class adjacent street, from 1 (collector with low traffic volume) to 3 (principal arterial with high traffic volume).^5^ The resulting final data describe 1,641 blocks nested in 343 neighborhoods. Table 1 presents descriptive statistics for these data.

**TABLE 1:** Descriptive Statistics

Measure	Mean	SD	Min	Density	Max
**Neighborhood (*N *= 343)**					
Collective Efficacy (2003)	.00	1.00	−3.64	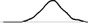	2.81
Collective Efficacy (1995)	.00	1.00	−2.93	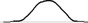	3.00
Disadvantage	.00	1.00	−2.35	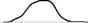	3.45
Stability	.00	1.00	−2.39	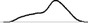	2.04
Hispanic/Immigrant	.00	1.00	−1.60	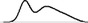	2.30
Density (Neighborhood)	7.10	4.38	.18	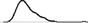	31.66
**Block (*N *= 1,641)**					
Homicide / Gun Assault	1.11	1.88	.00	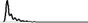	21.00
Robbery	3.18	4.39	.00	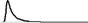	44.00
Violent Crime	6.42	8.34	.00	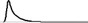	79.00
Property Crime	20.33	24.62	.00	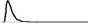	315.00
Abandoned	.12	.21	.00	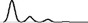	1.00
Bars	.05	.13	.00	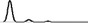	1.00
Commercial Dest.	.21	.26	.00	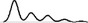	1.00
Liquor	.03	.10	.00	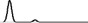	.75
Mixed Land Use	.32	.32	.00	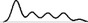	1.00
Parking	.11	.16	.00	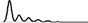	1.00
Recreation	.05	.09	.00	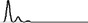	1.00
Vacant	.12	.21	.00	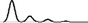	1.00
Street Class	1.83	.83	1.00	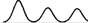	3.00
Density (Block)	10.85	7.59	.00	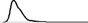	83.42

## 4 METHOD

**FIGURE 3:**
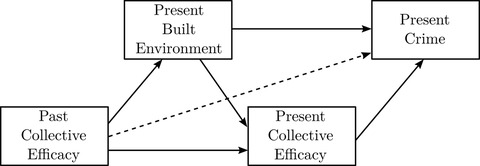
Longitudinal structural model *Note*: Dashed arrow represents tested independence restriction (*d*‐separation)

I examine the relationships between collective efficacy, the built environment, and crime using a piecewise structural equation model (SEM; see figure 3), which consists of 1) negative binomial mixed models predicting crime with collective efficacy and built environment characteristics, as well as 2) linear mixed models predicting present collective efficacy and built environment conditions using past collective efficacy. Piecewise SEM is an alternative to conventional covariance‐based SEM that instead decomposes the structural model into component regressions estimated separately (Shipley, 2016). This permits use of estimators that are computationally intractable or unsupported in conventional SEM software. In the present case, a piecewise approach permits mixing single‐ and multilevel linear and negative binomial models.

Because the component models are estimated individually, the fit of a piecewise SEM is evaluated using tests of directed separation (*d*‐separation) for each independence restriction in the system of models. In this case, the tests of directed separation are based on the significance of correlations between the residuals of endogenous variables and/or observed values of exogenous variables that the structural model implies should be zero (Shipley, 2016). *D*‐separation tests in the model are summarized by a Fisher's *C* statistic, which measures overall fit similar to chi‐square tests based on comparisons of observed and predicted covariance matrices in conventional SEM. Both the Fisher's *C* statistic and SEM chi‐square may be described as simultaneous tests of the validity of all restrictions implied by the structural models.

Figure 3 is a simplified version of the complete structural model. Each solid arrow represents a separate model or set of models testing the hypothesized causal pathway(s). The dashed arrow from past collective efficacy to crime is expected to be zero conditional on the included measures. This is evaluated using a test of *d*‐separation for each outcome. The entire system of models was estimated simultaneously using the R package piecewiseSEM (Lefcheck, 2016; R Core Team, 2021).

At the neighborhood level, all models adjust for prior (year 2000) neighborhood disadvantage, stability, Hispanic/immigrant concentration, and population density. At the block level, models adjust for population density rather than using a population offset to model rates as is often advised in models of crime counts (Osgood, 2000). This choice was made because it is likely block‐level populations capture variation in all three key elements of criminal opportunity—likely offenders, suitable targets, and capable guardians—which are unaccounted for by other covariates. Testing different functional forms of density revealed a strong quadratic relationship at the block level in all crime models, which might be expected if density captures both potential targets and guardians: Crime is more likely to occur where there are sufficient people present to make targets abundant but not so many as to make it likely the crime will be observed or interrupted (Angel, 1968; St. Jean, 2007, p. 156). Inclusion of the density measures is also conservative as removing them strengthens the focal relationships. Block‐level models also include a street class measure to partly control for accessibility that influences criminal opportunity (Johnson & Bowers, 2010). These measures do not fully account for ambient population, but ambient population is a mechanism—a mediator—for the effects of some built environment features. Controlling for local foot traffic that is the result of the features of interest will induce post‐treatment bias.

Spatial effects of collective efficacy and built environment features are commonly found in the literature (e.g., Bernasco & Block, 2011; Groff & Lockwood, 2014; Morenoff et al., 2001). The present research design cannot examine spatial effects at the block level because nearly all sampled blocks are nonadjacent (see figure 2 above). The present analyses are thus limited to estimating local, within‐block associations between built environment features and crime. Block faces surrounding the focal block are included in the built environment measures, however, which may account for some spatial spillover from adjacent blocks. Lack of adjacency also precludes testing for spatial dependence using neighbor matrices (e.g., Moran's *I*). No evidence was found for spatial dependence using semivariance analysis of block centroids, however, and no significant effects were found for spatial lags of neighborhood‐level independent variables.

### 4.1 Models of crime

**FIGURE 4:**
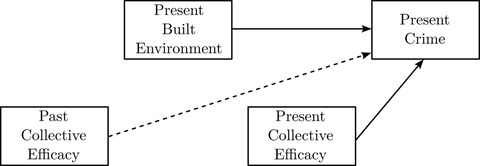
Simplified depiction of crime models *Notes*: Solid arrows are modeled direct effects. The dashed arrow represents unmodeled (restricted) paths

Hypothesis 1 proposes there is a conditional direct association between built environment features and specific types of crime based on the form of opportunities they provide—for instance, I expect commercial destinations to better predict robbery and property crime than homicide and gun assaults. Although commercial destinations may promote crime of all kinds by bringing many people together, they in particular feature suitable targets for theft (merchandise) and robbery (customers carrying cash). Figure 4 is a simplified diagram of the model focusing on the measures of interest. Note that the built environment box represents all eight built environment features—bars, liquor stores, vacant lots, abandoned buildings, commercial destinations, recreation facilities, parking lots, and mixed land use—and crime represents all four crime types—homicide and gun assaults, robbery, any violent crime, and any property crime. In all cases I expect a direct effect of collective efficacy on crime as a result of the mechanism of informal social control. The dotted line indicates restrictions evaluated using tests of *d*‐separation.

I estimate the conditional direct effects of collective efficacy and the built environment on crime using negative binomial models with random intercepts for neighborhoods fit using R's lme4 package (Bates et al., 2015). Neighborhood intercepts address correlations in residuals for blocks in the same neighborhood. Conditional on the included covariates, the intraclass correlations are modest (between .10 and .20 depending on crime type); however. BIC values and likelihood ratio tests indicate specifications with the random effects are at least weakly preferred.

### 4.2 Models of the built environment and present collective efficacy

**FIGURE 5:**
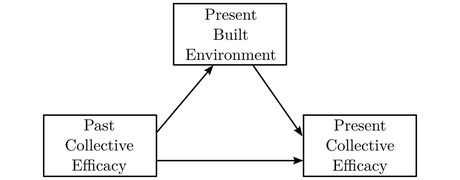
Models of the built environment and present collective efficacy

The next set of models estimate the conditional direct associations between past collective efficacy and the built environment, as well as between both past collective efficacy and the built environment and present collective efficacy. Hypothesis 2 posits that past collective efficacy influences the built environment, and hypothesis 3 posits that features of the built environment impact present collective efficacy. The solid arrows in figure 5 depict the tested relationships.

This part of the piecewise structural model consists of pooled and multilevel linear regressions. A pooled (neighborhood‐level) linear regression was used to test the paths from the built environment and past collective efficacy to present collective efficacy. Multilevel (block‐in‐neighborhood) linear regressions test the paths from past collective efficacy to the built environment features. As before, all models adjust for neighborhood structural characteristics, and the built environment features are permitted to correlate with each other. Note that the neighborhood structural characteristics were measured in the year 2000 and past collective efficacy was measured in 1995. If collective efficacy influences any built environment features via these structural characteristics, this amounts to controlling for a post‐treatment confounder (a mediator). Consequently, this may yield conservative estimates of the relationships between past collective efficacy and both the built environment and present collective efficacy. An alternative specification modeling these mediation pathways produced substantively equivalent results.

## 5 RESULTS

This section presents results from each set of models described above. The first subsection, Crime results, provides estimates for the conditional direct associations of collective efficacy, the built environment, and tract‐ and block‐level controls with the four forms of police‐reported crime. The second subsection, Built environment and collective efficacy results, provides estimates of the associations between past collective efficacy and the built environment and between the built environment and present collective efficacy.

### 5.1 Crime results

**FIGURE 6:**
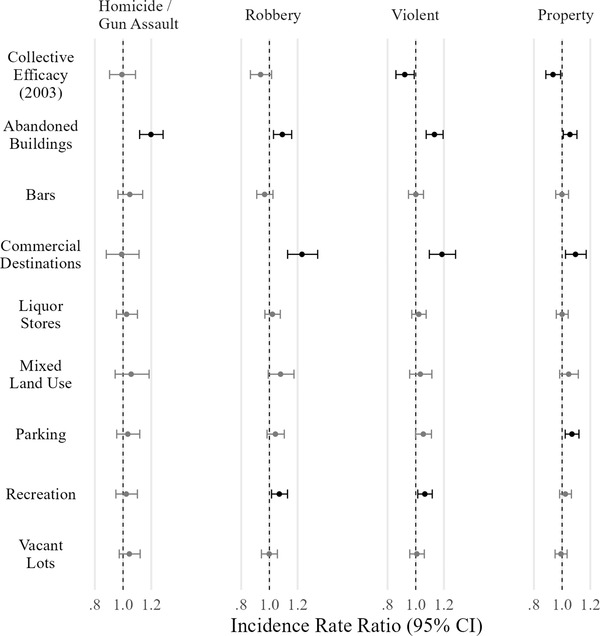
Estimated incidence rate ratios and 95% confidence intervals for selected predictors of five crime types *Notes*: Predictors are standardized, and outcomes are log‐counts. Estimates significant at *p* < .05 are in black. *N* = 1,641

Figure 6 displays incidence rate ratios (IRRs) for the conditional direct associations between the primary predictors of interest—collective efficacy and the built environment features—and crime. Each column represents a model for a different crime type. The displayed IRR is the estimated multiplicative difference in the count of crime incidents for a 1 standard deviation difference in the predictor. For example, a 1 standard deviation higher level of abandoned buildings—21 percent more block faces with abandoned buildings on and around that block—is expected to be associated with, on average, approximately 20 percent more homicides and gun assaults than an otherwise similar block.

The primary result seen in figure 6 is that the conditional direct associations of some built environment features with crime are large as proposed in hypothesis 1. Homicide and gun assaults are significantly predicted only by abandoned buildings. All violent crimes, robbery, and property crimes are also associated with abandoned buildings, although less so. Features that provide targets of monetary value—commercial destinations and parking—are associated with robbery and property crime. Commercial destinations and potentially parking and mixed land use show similar associations with robbery and all violent crime as well, however.^6^ As noted before, this is consistent with routine activities theory if these built environment features are associated with increased foot traffic, which increases the potential for interpersonal interactions of any kind, including violent ones.

Against hypothesis 1, vacant lots, bars, and liquor stores show no significant relationship with crime. This may be a result of treatment heterogeneity: The measures do not distinguish between different types of establishments or vacant lots. It is possible, for example, that abandoned buildings are nearly always suitable for concealing weapons but only particular vacant lots are suitable—such as those with substantial debris or foliage. Similarly, it is likely certain bars provoke violence—as a result of service practices, property management, or clientele—whereas others do not, or even reduce it through monitoring and reporting of problems (Graham et al., 2006). The present research design cannot examine potential heterogeneity of this sort. Appendix 3 in the online supporting information examines the possibility that the effects of built environment features are moderated by collective efficacy—which might capture some heterogeneity—but I find little evidence for this proposition.

In contrast to expectations, the direct association between present collective efficacy and crime is small in magnitude conditional on block‐level covariates and structural neighborhood characteristics. Estimated IRRs for collective efficacy are near zero for homicide and gun assault and significant only for violent and property crimes—but similar in magnitude to robbery. This is striking as past research using similar research designs typically has found a negative relationship of collective efficacy on crime (Lanfear et al., 2020), including one analysis using the same CCAHS data, although pooled with the 1995 PHDCN data and aggregated to tracts instead of neighborhoods (Sampson, 2012, pp. 173−177). It is possible that the block‐level measures are in part capturing heterogeneity in collective efficacy at the neighborhood level (e.g., Weisburd et al., 2020). Removing the built environment features does not notably strengthen the estimates, although an alternative model featuring block‐level collective efficacy (in many cases calculated from a single respondent) reveals a modest relationship with gun violence and assault (–.07, *p* = .046). Results were otherwise unchanged, however.^7^ The weak estimate for 2003 collective efficacy may instead be from attenuation resulting from the smaller sample size in the 2003 survey (3,074), as compared with the 1995 survey (7,672), which yields less reliable estimates of collective efficacy (.50 vs. .76). Appendix 4 in the online supporting information describes sensitivity tests that suggest that even though the estimated effect of 2003 collective efficacy is likely attenuated by unreliability, it is substantially weaker than would be expected from smaller neighborhood sample sizes alone. Given the potential for attenuation, however, the present models should not be considered strong tests of the effect of contemporaneous collective efficacy on crime.

For reference, the full model estimates are found in table 2. These estimates are the log‐count marginal effects on crime from 1 standard deviation differences in predictors. That is, the value of .69 for the Disadvantage row in the Homicide/Gun Violence column indicates a 1 standard deviation higher level of disadvantage is associated with a .69 higher log‐count of homicides on a given block. The IRR estimates in figure 6 are exponentiated values of these estimates. The nonsignificant *d*‐separation test *p*‐values at the bottom of the table indicate no association was found between past collective efficacy and any of the crime outcomes net of included covariates (Overall Fisher's C=12.1, df=8, p=.15). This result is consistent with the expectation that past collective efficacy exerts no protective effect on crime except via present collective efficacy or the built environment.

**TABLE 2:** Negative Binomial Regression Estimates of Crime

**Predictor**	**Homicide/Gun Assault**	**Robbery**	**Violent**	**Property**
**Neighborhood**				
Coll. Eff (2001)	−.01(.05)	−.06(.04)	**−.08(.04)**	**−.07(.03)**
Disadvantage	**.69(.05)**	**.22(.04)**	**.38(.04)**	**−.09(.03)**
Stability	−.03(.06)	**.15(.05)**	**.13(.04)**	**.29(.03)**
Hispanic/Immigrant	**−.16(.05)**	**−.40(.04)**	**−.31(.03)**	**−.22(.03)**
Density (Neighb.)	.07(.05)	**.25(.04)**	**.17(.04)**	**.09(.03)**
**Block**				
Abandoned	**.18(.04)**	**.09(.03)**	**.12(.03)**	**.05(.02)**
Bars	.05(.04)	−.03(.03)	.00(.03)	.00(.02)
Commercial Dest.	−.01(.06)	**.21(.04)**	**.17(.04)**	**.09(.03)**
Liquor Stores	.02(.04)	.02(.03)	.02(.03)	.00(.02)
Mixed Land Use	.06(.06)	.08(.04)	.03(.04)	.05(.03)
Parking	.03(.04)	.04(.03)	.05(.03)	**.07(.02)**
Recreation	.02(.04)	**.07(.03)**	**.06(.02)**	.02(.02)
Vacant	.04(.04)	.00(.03)	.01(.03)	−.01(.02)
Street Class	**.17(.04)**	**.23(.03)**	**.19(.03)**	**.14(.02)**
Density (Block)	**.15(.06)**	**.13(.04)**	**.21(.03)**	**.12(.03)**
Density (Block)^2^	**−.37(.08)**	**−.10(.03)**	**−.14(.03)**	**−.05(.02)**
Past Coll. Eff. *d*‐Sep. *p*‐value	.15	.69	.06	.39
*R* ^2^	.20	.30	.37	.30

*Notes*: N = 1,641 for all models; Standard errors in parentheses; Trigamma R2.

Bolded estimates significant at 95% level.ablef.

It is also noteworthy that property crimes display weak and negative relationships with disadvantage, unlike the other forms of crime that are positively related to disadvantage. This may be indicative of the availability or value of targets for property crime in more structurally advantaged areas, or perhaps differential rates of reporting across neighborhoods. Block‐level population density also exhibits a parabolic relationship with crime in all models. Under the strong assumption block population density captures the average number of people in the area at any given time, this may be evidence for the aforementioned opportunity trade‐off between the number of available targets and capable guardians. The consistently large estimates for the street class control are possibly indicative of criminogenic effects of easier access or larger ambient populations as well.

### 5.2 Built environment and collective efficacy results

The next models test the second hypothesis—past collective efficacy reduces criminogenic features of the built environment—and the third hypothesis—criminogenic features of the built environment reduce present collective efficacy. Together, the models used to test hypotheses 2 and 3 form the first stage estimates of the structural model in figure 3. Table 3 depicts the estimates obtained from the piecewise structural equations. Units of both predictors and outcomes are standardized, so coefficients may be interpreted as expected standard deviation differences in the outcome (heading measure) given 1 standard deviation differences in the predictor (left margin measure). Standard errors are in parentheses. Coefficients significant at p<.05 are bolded. *R* ^2^ values are conventional for collective efficacy (single‐level model) and marginal for all others.

**TABLE 3:** Linear Regression Estimates of Built Environment Features and Collective Efficacy

	Collec.	Aband‐		Commer.	Liquor	Mixed			
Predictor	Effic.	oned	Bars	Dest.	Stores	Land Use	Parking	Recreation	Vacant
**Neighborhood**									
Coll. Eff. (1995)	**.24(.03)**	**−.11(.04)**	−.04(.03)	**−.07(.04)**	−.02(.04)	**−.09(.03)**	−.01(.04)	.03(.04)	**−.14(.05)**
Disadv.	**−.14(.03)**	**.32(.03)**	**−.17(.03)**	**−.11(.03)**	−.01(.03)	**−.11(.03)**	−.05(.04)	−.06(.04)	.00(.04)
Stability	**−.20(.03)**	.00(.04)	**.10(.03)**	**.13(.03)**	**.10(.03)**	**.13(.03)**	**.20(.04)**	**.24(.04)**	.05(.04)
Hispanic/Immigrant	.04(.02)	**−.18(.03)**	**.17(.03)**	**.17(.03)**	−.01(.03)	**.21(.03)**	−.04(.03)	−.06(.03)	.07(.04)
Density (Neighb.)	**−.07(.03)**	−.02(.04)	−.04(.03)	−.02(.03)	.03(.04)	−.03(.03)	.06(.04)	−.05(.04)	−.07(.05)
**Block**									
Abandoned	−.05(.02)	—	—	—	—	—	—	—	—
Bars	.02(.02)	—	—	—	—	—	—	—	—
Commer. Dest.	.00(.04)	—	—	—	—	—	—	—	—
Liquor Stores	.04(.02)	—	—	—	—	—	—	—	—
Mixed Land Use	−.05(.03)	—	—	—	—	—	—	—	—
Parking	.04(.02)	—	—	—	—	—	—	—	—
Recreation	−.02(.02)	—	—	—	—	—	—	—	—
Vacant	−.02(.02)	—	—	—	—	—	—	—	—
Street Class	.00(.02)	.04(.02)	**.12(.02)**	**.34(.02)**	**.16(.02)**	**.40(.02)**	**.24(.02)**	**.07(.02)**	**.13(.02)**
Density (Block)	**−.09(.03)**	−.03(.03)	−.01(.03)	**.09(.03)**	.01(.03)	**.09(.03)**	−.04(.03)	.01(.03)	.01(.03)
Density (Block)^2^	.01(.02)	.00(.02)	−.01(.03)	−.05(.02)	−.02(.03)	−.01(.02)	**.06(.02)**	.00(.03)	−.02(.02)
*R* ^2^	.30	.22	.08	.22	.05	.28	.11	.05	.05

*Notes*: N = 1,641 for all models. Standard errors in parentheses.

Bolded estimates significant at 95% level.

We see here that present collective efficacy is mainly predicted by past collective efficacy, stability, and disadvantage. In partial support of hypothesis 2, collective efficacy appears to be one of the primary predictors of abandoned buildings, mixed land use, vacant lots, and commercial destinations. These associations are notable as abandoned buildings are a strong predictor of homicide and gun assault, and commercial destinations are important predictors of robbery, property crime, and general violence. In evidence against hypothesis 3, none of the built environment features significantly predict present collective efficacy net of the other covariates. This may in part be the result of imprecise estimates as a result of low reliability in 2003 collective efficacy.

As noted earlier, it is unlikely the present modeling approach satisfies the sequential ignorability assumption necessary to identify the mediated causal effect of past collective efficacy on crime via the built environment (Imai et al., 2010). Estimates of these mediated pathways may still be of interest as illustrative results. If we assume the structural model is correctly specified, the estimated indirect effect on block counts of crimes from a one standard deviation higher level of collective efficacy (based on the IRR) is 1.5 percent for property crime and between 2.7 percent and 3.1 percent for all other types. For homicide and gun assault, nearly all of the protective indirect association (3.1 percent) is attributable to reductions in abandoned buildings and mixed land use (2.5 percentage points). These indirect associations may appear modest, but they are on average three times the magnitude of the direct associations between present collective efficacy and crime.

## 6 DISCUSSION

The primary finding in this work is that criminogenic built environment features are associated with past collective efficacy, suggesting collective action might affect crime by altering the physical environment. Abandoned buildings and commercial properties appear particularly important. If these results are robust, the existence of a collective efficacy crime control pathway via the built environment is important because changes to the environment do not require continued intervention, thus, making them stable and low cost to residents (MacDonald et al., 2019). This may reinforce informal social control over time, indirectly, via the established negative effect of crime on collective efficacy (Sampson, 2012). Future research should attempt to replicate this result in other settings, as well as investigate the proposed mechanism—influence over local government agencies and policymakers—which could not be examined with the present research design.

No evidence was found, however, for a direct influence of these built environment features on collective efficacy net of neighborhood structural measures. It is possible, however, that collective efficacy is responsive to changes in the built environment features rather than levels. For example, perhaps resident confidence in their ability to solve problems is bolstered by declines in abandoned buildings and other problem properties, regardless of the overall number. Conversely, even at low levels of abandoned buildings, an increase of one or two abandoned buildings might be interpreted as a sign the neighborhood is in decline and out of control (Wilson & Kelling, 1982). This cannot be tested with the present data but should be considered in future research.

This relationship between collective efficacy and the built environment may have also implications for the stratification of neighborhoods within a metro area. The literature on the political economy of place and public social control tells us that differences in the ability to regulate the built environment contribute to race and class stratification (Logan & Molotch, 2007). For example, researchers have found collective action in affluent White neighborhoods, mainly via local government, helps maintain housing segregation and concentrate public housing in poor neighborhoods (Einstein et al., 2020). The ability of one neighborhood to exert control over its space can thus produce metro‐wide consequences. This may foster the concentration of disadvantage, and thus crime, implicating collective efficacy in the process.

Although this work focused on crime as an outcome, the reach of collective efficacy suggests a wider vision for the built environment as a mechanism. Collective efficacy is a general problem‐solving capacity associated broadly with community well‐being (see Sampson, 2012, pp. 159−161 for a review). Even though I demonstrate negative associations between past collective efficacy and some criminogenic features likely perceived as problematic by residents—in particular abandoned buildings—it is reasonable to expect an opposite effect for features of the built environment that promote well‐being. Collectively efficacious neighborhoods may, as the indicator suggests, be more effective at preserving a library or fire station threatened by budget cuts. In this way collective efficacy may generally foster the development, maintenance, and improvement of built environment features that produce use value in neighborhoods (Logan & Molotch, 2007). This could not be tested with the present data, but it is an important avenue for future research.

The converse of this, of course, is that low collective may result in disadvantaged neighborhoods accumulating problematic features and losing beneficial ones, including public infrastructure. When governmental and institutional disinvestment occurs, the effects are more likely to be concentrated in neighborhoods unable to mount effective campaigns to maintain services. This may be particularly painful when communities face closures of beneficial local facilities and services, yet receive stable or even increasing levels of law enforcement scrutiny (Beck & Goldstein, 2018). Interventions in the built environment are a promising alternative to increased policing for addressing crime in disadvantaged neighborhoods, particularly serious violence (Kondo et al., 2018). Remediation of criminogenic features of the environment is often inexpensive, effective, and politically feasible—and generates benefits beyond crime control (MacDonald et al., 2019). For example, Branas et al. (2018) found vacant lot remediation increased resident outdoor socializing and reduced fear of victimization. Substantial reductions to crime and improvements to well‐being could be made in disadvantaged neighborhoods using programs that work with communities to address problematic built environment features—and create or improve beneficial ones.

Although these results are suggestive, this approach does not conclusively establish a causal relationship nor provide evidence for the proposed mechanism of influencing institutions affecting property development. Ideally, stronger tests of these relationships and mechanisms would be conducted using longitudinal designs and field experiments. This is, however, a challenging target for quantitative research as a result of the combination of slow change in the built environment and the interdependence of social and physical characteristics of neighborhoods. These relationships and mechanisms may be more amenable to qualitative or mixed‐method approaches examining collective action to alter the built environment for crime control purposes. This might include observation of public meetings—such as of zoning boards—as well as analyses of meeting records and media reports of protests, legal actions, and direct interventions (e.g., Einstein et al., 2020). Analyses linking rich qualitative data to existing quantitative data on neighborhood collective efficacy, the built environment, and crime are likely to be illuminating.

Another limitation of these analyses is that they cannot strongly test the moderating effects of neighborhood context on the associations between built environment characteristics and crime (see appendix 3 in the online supporting information). It is a common finding that the effects of built environment features on crime are moderated by community social structure (Tillyer et al., 2021; Wilcox et al., 2003; Wilcox & Tillyer, 2017). In other cases, the strongest evidence for built environment effects comes from studies restricted to disadvantaged contexts. As an example, remediation experiments that found the strong effects of vacant lots on violent crime were conducted primarily in poor, high‐crime neighborhoods (Branas et al., 2018). These effects may be weaker in less disadvantaged contexts. Importantly, if certain features increase crime only under particular conditions, one would expect residents to work to remove them only under those same conditions unless they are otherwise problematic (e.g., threatening property values). Stronger examinations of multilevel interactions are an obvious next step but require more statistical power than the present data permit, particularly given the rarity with which features like abandoned buildings are found in advantaged neighborhoods. Survey data with a larger sample size per unit, particularly at smaller units, would result in more reliable collective efficacy measurements and, thus, in a more convincing test of its effects, both direct and as a moderator. This would also facilitate research disentangling effects of collective efficacy at different spatial scales (Boessen & Hipp, 2015; Hipp, 2007; Hipp & Boessen, 2013), which may be important if collective efficacy's direct effects are more local (e.g., Weisburd et al., 2020) but indirect effects, such as changing the built environment, have a longer reach.

In a related vein, more refined measures may be needed to accurately estimate relationships between built environment features and crime. A weak relationship between alcohol outlets or vacant lots and crime may be a result of heterogeneity in social meaning and function of these places, or differences in reporting behavior rather than underlying rates of crime. For alcohol outlets in particular, these results may reflect differences in management, with some well‐regulated and others not (Graham et al., 2006). Efficacious place management is a major source of heterogeneity of criminogenic effects between otherwise similar places (Eck & Madensen, 2018). The present analysis treats bars that engage in over‐service and turn a blind eye to illicit activity as equivalent to well‐regulated ones, and it treats all vacant lots as similar, whereas their true effect is likely contingent on the concealment they provide. This calls for better measures, for example distinguishing between bars using business descriptions or administrative records like liquor violations.

As a result of an absence of direct measures of ambient population, this analysis may also treat busy properties as equivalent to low‐traffic ones. Even though the present study cannot test this hypothesis, it is likely much of the criminogenic effects of built environment features—and heterogeneity within features—is a result of ambient populations (Wilcox & Eck, 2011). Although not available in the time period under examination, this could be addressed in future studies using digital trace data on human mobility (e.g., Saxon, 2021; Tucker et al., 2021). Some portion of this heterogeneity may also be captured using moderation models if ambient populations—or other unobserved characteristics of places—are related to neighborhood social structure. For example, collective efficacy may moderate built environment effects by governing the movement of nonresidents—even by imposing physical impediments (Donnelly & Kimble, 1997). Research examining this heterogeneity should be pursued for all built environment features using data on human mobility at small geographic areas. Data on spatially contiguous units would additionally permit analyzing spatial spillover effects that could not be estimated in the present study.

In a broader sense, social structure, the built environment, human mobility, and crime are interwoven in the city—a point long recognized in urban studies (Jacobs, 1961; Suttles, 1968; see also Browning et al., 2021). Recent advances, such as those using digital trace data (e.g., Levy et al., 2020; Saxon, 2021) and ecological networks based on surveys and simulation (e.g., Browning, Calder, Boettner, et al., 2017; Browning, Calder, Soller, et al., 2017), are rapidly improving our understanding of the role of mobility in this system. The present study examined how social structure might influence crime via the built environment, leaving mobility an implied but unanalyzed mechanism. Future research might unite these approaches to specify mobility as both a cause and a consequence of macro‐level social structures (e.g., collective efficacy) and micro‐level situations (e.g., crime) embedded in a changing built environment (see Lanfear, 2021).

Different forms of social capital—such as reciprocated exchange or intergenerational closure—may also be more relevant than social cohesion or control expectations for controlling the built environment. Similarly, it is likely that resources such as legal expertise—which may be inconsequential for informal social control—are important predictors of collective efficacy for these tasks (e.g., Einstein et al., 2020). This might be addressed in future surveys by including questions about the perceived capacity of residents to engage in legal or political challenges.

Finally, changing the built environment often requires working through institutions that may be unresponsive or even hostile—particularly to neighborhoods that are disadvantaged or have large BIPOC populations. Future research would benefit from task‐specific measures of collective efficacy that capture resident expectations for the responsiveness of actors, such as public officials. This responsiveness likely differs by metropolitan context. For example, Chicago, the city under study, may be a unique context for citizen–government interactions. On the one hand, the city may be more responsive to residents as a result of its decentralized system of governance, in which each of the city's 50 wards elects an alderman to the legislative body. Residents frequently work through these aldermen or their appointees to influence city government to address crime (Carr, 2005; Vargas, 2016). On the other hand, this system of government is also characterized by political competition that inhibits the ability of neighborhoods to fight serious crime (Vargas, 2016). In other cities, policy makers may be more or less responsive to the demands of residents—or the demands of developers and owners of properties residents perceive as problematic. Criminogenic effects resulting from poor management by property owners may be elevated where owners feel little pressure from a city government unresponsive to residents (Eck & Madensen, 2018). Data from other cities, or, better, from a multicity sample, should be used to examine whether this study's findings are replicable in different contexts of local government.

Despite these limitations and outstanding questions, I believe this analysis makes an important contribution to the literature on neighborhood crime control. The theoretical framework presented suggests a new mechanism by which collective efficacy may shape neighborhood crime rates—control of the built environment. Despite often being used to operationalize only informal social control, collective efficacy has been conceived of as a general problem‐solving capacity of neighborhood residents (Sampson, 2012). Rather than just promoting guardianship by residents, such as monitoring or direct intervention, collective efficacy may also reduce crime by empowering residents to remove or prevent the development of sources of criminal opportunities. This crime control pathway is important because changes to the built environment are long‐lasting and reduce the need for future resident interventions against crime. Control of the built environment has implications beyond crime as well, as the built environment is a major factor governing the quality of life and well‐being of residents (Logan & Molotch, 2007; MacDonald et al., 2019). This may be an important mechanism by which collective efficacy promotes stable, safe, and livable neighborhoods.

## Supporting information



Online Appendix

## Footnotes

1 Additional supporting information can be found in the full text tab for this article in the Wiley Online Library at http://onlinelibrary.wiley.com/doi/10.1111/crim.2022.60.issue‐2/issuetoc.

2 Two neighborhoods are unusually large at greater than 10 square miles each, as a result of the inclusion of large open areas: O'Hare Airport and Lake Calumet.

3 The 1990 factor scores calculated without this indicator exhibit correlations greater than .95 with those from Sampson et al. (1997).

4 Two blocks in the sample had zero population reported in the 2000 decennial census.

5 Only 1 percent of blocks featured other categories (e.g., freeway) as their most major adjacent street. These were collapsed into adjacent main categories. Disaggregated and categorical (dummy variable) specifications were rejected as a result of worse fit by large (> 10) BIC differences. Primary estimates were unchanged across specifications.

6 I note the result for mixed land use because the estimate is affected by a high correlation with commercial destinations (.75). Similar, but more precise, results are obtained for both predictors by omitting the other.

7 Estimates were also robust to introducing tract‐level, social‐structural variables, block area, alternative functional forms of controls (e.g., splines), and a control for prior neighborhood‐level homicide.
