# Supplementary material for: Collective efficacy and the built environment*
Source: Criminology. 2022 Feb 15;60(2):370–96. doi: 10.1111/1745-9125.12304 (PMC9303720; doi:10.1111/1745-9125.12304)
Supplement: Supplementary file 1 — Online Appendix [file CRIM-60-370-s001.docx]

SUPPORTING INFORMATION FOR

“COLLECTIVE EFFICACY AND THE BUILT ENVIRONMENT”^[[1]](#footnote-1)^*

CHARLES C. LANFEAR

Appendix

# Neighborhood Social and Perceptual Measures

Collective efficacy and its subscales (informal control expectations and cohesion and trust) were calculated using multilevel measurement models that adjust for sociodemographic composition of neighborhoods and shrink estimates toward the grand mean where interrater reliability is lower (Raudenbush et al., 1991; Sampson et al., 1997). All indicators are four- or five-category ordinal indicators estimated using linear regression with dummies for each indicator to adjust for item difficulty and random respondent intercepts. All models feature neighborhood-level random intercepts that are extracted and used in subsequent models as empirical Bayes estimates of the neighborhood measures. I use a single factor for collective efficacy that combines cohesion and trust and informal control expectations to mimic existing work in this field and using these data (e.g., Sampson et al., 1997). A two-factor solution is weakly preferred in confirmatory factor models, although the resulting separate factors are highly correlated at both the individual ($\rho=.70$ in 1995, $\rho=.67$ in 2003) and the neighborhood level ($\rho=.71$ in 1995, $\rho=.64$ in 2003).

Table A1 depicts all indicators used to calculate the neighborhood measures including loadings from confirmatory factor analyses and average neighborhood-level reliability (not within-individual reliability) from each multilevel model.^[[2]](#footnote-2)^ Bracketed loadings reflect loadings on separate cohesion and trust and informal control expectations factors for comparison with the combined collective efficacy factor.

# Neighborhood Structural Measures

The social disorganization tradition recognizes crime and social control are rooted in structural characteristics of neighborhoods. To construct these measures, I obtained nine decennial census measures from the Longitudinal Tract Data Base (LTDB; Logan et al., 2014) based on those used by Sampson et al. (1997). The LTDB reweights indicators across changing boundaries between the 1990 and 2000 censuses to ensure they describe the same geographic areas. All measures are percentages and are listed in table A2. I use an oblimin-rotated, alpha-scoring factor analysis to perform dimension reduction on these measures (Kaiser & Caffrey, 1965). The factors were calculated simultaneously using both 1990 and 2001 observations for each NC to generate comparable measures over time. Three factors explain 87 percent of variation in the nine indicators and exhibit acceptable model fit ($\chi^{2}=7.69$, $df=12$, $p=.81$). Loadings are depicted in table A2.

Based on the indicators that most highly load on each factor, they are described as disadvantage, Hispanic/immigrant (concentration), and stability. Disadvantage loads primarily on the 18 years old or younger population, unemployment, poverty, and female-headed households, as well as to a lesser degree on the non-Hispanic Black population. Hispanic/immigrant loads on Hispanic population, foreign born, and negatively on non-Hispanic Black. Stability loads negatively on home ownership and positively on moves in the last 10 years. These were extracted as neighborhood-level, correlation-preserving factor scores to produce a parsimonious set of structural predictors for use in subsequent models (ten Berge et al., 1999).

Although the measures were chosen to replicate the structural measures used in Sampson et al. (1997), one indicator for disadvantage—percentage of families on public assistance—was not available in the LTDB. The indicator for recent moves is also in the last 10 years rather than 5 years. Even with these differences—and calculating across both waves simultaneously—the resultant factor scores for 1990 neighborhoods are all correlated at over $\rho=.95$ with those from Sampson et al. (1997).

# Built Environment Moderation

Early intervention in the built environment may have the added benefit of reducing future social control burdens. The absence of criminogenic features may explain low crime in areas lacking collective efficacy. Conversely, the presence of criminogenic features may make crime “sticky” even in the presence of concerted action by residents (St. Jean, 2007). Therefore, collective efficacy could be more effective at restraining crime in the absence of environmental features that inhibit the exercise of informal control. Consequently, one might expect characteristics of the built environment to moderate the effect of collective efficacy on crime (see Wilcox & Tillyer, 2017).

This alternative set of models tests whether features of the built environment moderate the association between collective efficacy and crime. These models mirror the prior models of crime but introduce interaction terms between collective efficacy and the built environment features. Based on my theoretical framework, I expect all interactions between collective efficacy and built environment characteristics to be positive because criminogenic features of the environment will attenuate the negative effect of informal social control on crime.

## Crime and Moderation of Collective Efficacy Results

The last set of models augments the original set examining associations with crime using interaction terms between collective efficacy and each hypothesized criminogenic built environment feature. Interaction terms permit evaluating whether these features present challenges—ecological disadvantage in St. Jean’s (2007) terms—which are resistant to social control efforts, that is, locations where the returns to collective efficacy are low. Given the weak associations between collective efficacy and each form of crime, and the modest sample size that results in underpowered tests of interaction, these results should be interpreted with caution. Only very strong relationships are likely to be detected, but they may also be the result of sampling error and excessive partitioning of limited variation.

Table A3 contains point estimates from the negative binomial models of crime with interactions between collective efficacy and the built environment features. As this table reveals, three interaction terms are significant but only one is of large magnitude—abandoned buildings for homicide and gun assault. These three interactions are all positive as expected, but under multiple testing, one would expect to obtain a similar number of significant interactions as a result of chance; this finding is not particularly noteworthy. Interactions are notorious for making strong demands of the data, in terms of statistical power, so it is unsurprising that little is found here. Similar results, although opposite in sign, are found interacting built environment features with disadvantage.

Nonetheless, these results may be indicative of violent crime in higher collective efficacy neighborhoods being concentrated in a small number of locations with abandoned buildings. As an examination of this, a cross-tabulation of homicide and gun assaults (not shown) reveals that in neighborhoods in the top quartile of collective efficacy, more than 58 percent of these events occur on blocks in the top quartile of abandoned buildings. In neighborhoods in the bottom quartile of collective efficacy, this value is only 28 percent. That is, in low collective efficacy neighborhoods, gun assaults are fairly evenly distributed across blocks regardless of the concentration of abandoned buildings. In high collective efficacy neighborhoods, gun assaults are found where abandoned buildings are concentrated. This relationship might reflect what St. Jean (2007, p. 220) described as efforts by efficacious residents to limit serious crime to particular areas of their neighborhoods because of the inability to eliminate it entirely.

# Reliability of 2003 Collective Efficacy

The estimated effect of 2003 collective efficacy on present crime in the structural models is weaker than typically found in the literature (e.g., Sampson et al., 1997). This may be attributable to low neighborhood-level reliability in 2003 collective efficacy (.50). Note that this is neighborhood-level reliability, not individual-level reliability—within individuals, the 1995 and 2003 scales exhibit similar performance in confirmatory factor models (see appendix 1). Neighborhood sample size impacts neighborhood reliability by affecting within-neighborhood variance (error variance at the neighborhood level). The equation for the reliability of neighborhood collective efficacy ($\lambda_{k}$) is $\lambda_{k}=\tau_{\beta}/\left( \tau_{\beta}+V_{k} \right)$, where $\tau_{\beta}$ is the variance of parameter $\beta_{0}k$ (neighborhood collective efficacy) and $V_{k}$ is the error variance (Raudenbush & Bryk, 2002, p. 46). Given the present three-level model, the error variance is $V_{k}=\tau_{pi}/J_{k}+\sigma^{2}/\sum\left( n_{jk} \right)$, where $\tau_{pi}$ is the variance of the level 2 (individual) intercept, $J_{k}$ is the neighborhood sample size, and $\sum\left( n_{jk} \right)$ is the total number of responses to survey indicators ($n$) across all respondents ($j$) within neighborhood $k$ (Raudenbush & Bryk, 2002, p. 230; Raudenbush et al., 1991, p. 312). It can be seen then that $V_{k}$ is inversely proportional to $J_{k}$, the neighborhood sample size. Consequently, I conducted three sensitivity analyses that manipulate neighborhood sample size to examine whether estimates were robust to varying levels of collective efficacy reliability.

First, if the effects of collective efficacy are smaller and even nonsignificant for 2003 relative to 1995 solely because it has lower reliability resulting from the smaller neighborhood sample sizes, then the 1995 results should also be attenuated and even nonsignificant if 1995 collective efficacy is reestimated with neighborhood sample sizes comparable with the CCAHS. I tested this by reestimating 1995 collective efficacy using a random subsample of 40 percent of the respondents within each neighborhood ($N=2,937$ total), producing a measure with reliability of .49 (very close to the .50 of the CCAHS). I then used this low reliability 1995 measure in two models of neighborhood-level 1995 homicide and violence using the specification of Sampson et al. (1997). Coefficients were in both cases attenuated by approximately 25 percent but statistically significant ($p<.01$) and still more than double the magnitude of the estimated effect of 2003 collective efficacy on 2004 crime.

Second, if weak effects of 2003 collective efficacy on crime are caused by low reliability, then a reanalysis using a subsample of neighborhoods with high numbers of respondents may be more likely to detect an effect. I examined this by reestimating 2003 collective efficacy on a subsample of 200 neighborhoods with 8+ CCAHS respondents (the median count) to obtain a higher reliability (.62, a 22% increase). This reliability is not far short of the tract-level reliabilities (.68) in Sampson and Raudenbush (1999) where strong effects of collective efficacy on crime were found. The results from this higher reliability 2003 subsample were nearly indistinguishable from those using the original lower reliability measure.

Third, to supplement the subsample-based sensitivity tests, I conducted a simulation to examine the consequences of varying neighborhood sample sizes on estimates of the effect of collective efficacy on crime. For simplicity, I generated a two-level hierarchical data set of observations of collective efficacy with $K=343$ (neighborhoods) and $J_{k}=100$ (respondents in each neighborhood) using the variance parameters for collective efficacy from the two-level models in Sampson et al. (1999, p. 642): $\tau_{\beta}=.13$, $V_{k}=.99$. I then generated a neighborhood-level crime outcome with a –.50 correlation with the true neighborhood collective efficacy value, as the present 1995 collective efficacy measure displays a correlation of –.47 with the 1995 log homicide rate. From these simulated data, I drew random samples for each value of $J_{k}$ from 3 to 50 (that is, from 3 to 50 respondents per neighborhood), calculating for each set of samples the mean reliability across neighborhoods^[[3]](#footnote-3)^ and the level of attenuation in the parameter estimate and its *t*-ratio. The proportion of attenuation moving from the 1995 reliability (.76) to the 2003 (.50) reliability was 19 percent for the point estimate and 21 percent for the *t*-ratio. The correlation between the 2003 collective efficacy measure and 2004 homicide is –.18, approximately 62 percent weaker than in 1995, and a larger difference than that seen between the 1995 reliability and the reliability associated with a neighborhood sample size of 3 (.27). Equivalent results were obtained by varying the variance parameters between other plausible values from the literature, including estimates from the present study’s measurement models, and allowing the within-neighborhood sample sizes to vary around an overall mean sample size.

In summary, similar parameter estimates for the effect of collective efficacy on crime could not be obtained for 2003 and 1995 by manipulating only neighborhood sample size. Although it is likely the 2003 measure is subject to some attenuation, it is reasonable to conclude that the difference between effects of 1995 collective efficacy and 2003 collective efficacy is not a result of purely lower neighborhood-level reliability of the 2003 collective efficacy measure resulting from smaller neighborhood sample sizes.

# Replication Files

All files required to reproduce this article, including analyses, plots, tables, and the text itself, are available at [github.com/clanfear/built_environment_ce](file:///C:\Users\Matthew\Downloads\github.com\clanfear\built_environment_ce). Necessary restricted-access files are not included, but their sources are documented in the replication repository readme.

# References

Kaiser, H. F., & Caffrey, J. (1965). Alpha factor analysis. *Psychometrika*, *30*(1), 1–14.

Logan, J. R., Xu, Z., & Stults, B. J. (2014). Interpolating U.S. Decennial census tract data from as early as 1970 to 2010: A longitudinal tract database. *The Professional Geographer*, *66*(3), 412–420.

Raudenbush, S. W., & Bryk, A. S. (2002). *Hierarchical linear models: Applications and data analysis methods* (2nd ed). Sage.

Raudenbush, S. W., Rowan, B., & Kang, S. J. (1991). A multilevel, multivariate model for studying school climate with estimation via the EM algorithm and application to U.S. High-school data. *Journal of Educational Statistics*, *16*(4), 295–330.

Sampson, R. J., Morenoff, J. D., & Earls, F. (1999). Beyond social capital: Spatial dynamics of collective efficacy for children. *American Sociological Review*, *64*(5), 633.

Sampson, R. J., & Raudenbush, S. W. (1999). Systematic social observation of public spaces: A new look at disorder in urban neighborhoods. *American Journal of Sociology*, *105*(3), 603–651.

Sampson, R. J., Raudenbush, S. W., & Earls, F. (1997). Neighborhoods and violent crime: A multilevel study of collective efficacy. *Science*, *277*(5328), 918–924.

St. Jean, P. K. B. (2007). *Pockets of crime: Broken windows, collective efficacy, and the criminal point of view*. University of Chicago Press.

ten Berge, J. M. F., Krijnen, W. P., Wansbeek, T., & Shapiro, A. (1999). Some new results on correlation-preserving factor scores prediction methods. *Linear Algebra and Its Applications*, *289*(1-3), 311–318.

Wilcox, P., & Tillyer, M. S. (2017). Place and neighborhood contexts. In D. Weisburd & J. E. Eck (Eds.), *Unraveling the Crime-Place Connection: New Directions in Theory and Policy* (pp. 121–142). Routledge.

| TABLE A1 Loadings and reliabilities for neighborhood perceptual measures. | | | |
| --- | --- | --- | --- |
|  |  | **Loadings / Reliabilites (A)** | |
| **Measure/Indicator** | | **PHDCN (1995)** | **CCAHS (2003)** |
| **Collective Efficacy (Cohesion & Trust + Informal Control Expectations)** | | A=.758 | A=.503 |
| **Cohesion & Trust ("How much do you agree that...")** | | A=.763 | A=.453 |
|  | People around here are willing to help their neighbors. | .699 [.766] | .775 [.813] |
|  | People in this neighborhood can be trusted. | .726 [.795] | .824 [.881] |
|  | This is a close-knit neighborhood. | .660 [.707] | .624 [.646] |
|  | People in this neighborhood generally... get along with each other. | .515 [.558] | .752 [.799] |
|  | People in the neighborhood... share the same values. | .483 [.521] | .751 [.790] |
| **Informal Control Expectations ("How likely is it...")** | | A=.705 | A=.469 |
|  | If a group of neighborhood children were skipping school and hanging out on a street corner, how likely is it that your neighbors would do something about it? | .708 [.788] | .652 [.780] |
|  | If some children were spray-painting graffiti on a local building, how likely is it that your neighbors would do something about it? | .792 [.874] | .677 [.811] |
|  | If a child was showing disrespect to an adult, how likely is it that people in your neighborhood would scold that child? | .629 [.705] | .593 [.689] |
|  | If children were fighting out in the street, how likely is it that people in your neighborhood would stop it? | .623 [.681] | .556 [.644] |
|  | Neighborhood residents would organize to keep closest fire station open if it were to be closed down by city because of budget cuts. | .615 [.652] | .578 [.653] |
| 'A=' indicates neighborhood-level reliability; Brackets indicate loadings for separate factors for cohesion and trust and informal control expectations. | | | |

| TABLE A2 Factor loadings for neighborhood structural measures. | | | |
| --- | --- | --- | --- |
| **Measure** | **Disadvantage** | **Hispanic/ Immigration** | **Stability** |
| 18 Years Old or Younger | 1.03 | .25 | –.13 |
| Unemployment | .74 | –.33 | .14 |
| Poverty | .69 | –.15 | .43 |
| Female-Headed Households | .67 | –.44 | .27 |
| Home Ownership | –.09 | .02 | –.96 |
| Moved in Last 10 Years | –.19 | .34 | .79 |
| Hispanic | .30 | .93 | .05 |
| Foreign Born | –.12 | .83 | .15 |
| Non-Hispanic Black | .43 | –.71 | –.03 |
| Eigenvalue | 3.15 | 2.74 | 1.98 |
| Proportion of Variance Explained | .35 | .30 | .22 |

| TABLE A3 Negative binomial estimates of crime. | | | | | |
| --- | --- | --- | --- | --- | --- |
|  | **Predictor** | **Homicide/ Gun Assault** | **Robbery** | **Violent** | **Property** |
| **Neighborhood** | | | | | |
|  | Coll. Eff (2001) | –.03 (.05) | –.07 (.04) | **–.08 (.04)** | **–.06 (.03)** |
|  | Disadv. | **.68 (.05)** | **.21 (.04)** | **.38 (.04)** | **–.09 (.03)** |
|  | Stability | –.04 (.06) | **.15 (.05)** | **.12 (.04)** | **.28 (.03)** |
|  | Hispanic/ Immigrant | **–.16 (.05)** | **–.40 (.04)** | **–.31 (.03)** | **–.22 (.03)** |
|  | Density (Neighb.) | .07 (.05) | **.26 (.04)** | **.17 (.04)** | **.09 (.03)** |
| **Block** | | | | | |
|  | Abandoned | **.21 (.04)** | **.09 (.03)** | **.14 (.03)** | **.07 (.02)** |
|  | Bars | .05 (.04) | –.04 (.03) | .00 (.03) | .00 (.02) |
|  | Commercial Dest. | –.02 (.06) | **.23 (.05)** | **.18 (.04)** | **.09 (.04)** |
|  | Liquor | .03 (.04) | .02 (.03) | .02 (.03) | .00 (.02) |
|  | Mixed Land Use | .06 (.06) | .07 (.04) | .03 (.04) | .05 (.03) |
|  | Parking | .04 (.04) | .04 (.03) | .05 (.03) | **.07 (.02)** |
|  | Recreation | .02 (.04) | **.08 (.03)** | **.07 (.03)** | .02 (.02) |
|  | Vacant | .04 (.04) | –.01 (.03) | .00 (.03) | –.01 (.02) |
|  | Street Class | **.16 (.04)** | **.23 (.03)** | **.19 (.03)** | **.14 (.02)** |
|  | Density (Block) | **.14 (.06)** | **.14 (.04)** | **.21 (.03)** | **.12 (.03)** |
|  | Density (Block)^2^ | **–.38 (.08)** | **–.10 (.03)** | **–.13 (.03)** | **–.05 (.02)** |
|  | CE × Abandoned | **.10 (.03)** | .02 (.03) | **.05 (.03)** | .04 (.02) |
|  | CE × Bars | –.02 (.05) | –.03 (.04) | –.02 (.03) | –.01 (.03) |
|  | CE × Commercial Dest. | .01 (.06) | .09 (.05) | .06 (.04) | .04 (.04) |
|  | CE × Liquor | .01 (.04) | –.03 (.03) | –.01 (.03) | –.02 (.02) |
|  | CE × Mixed Land Use | .00 (.06) | .00 (.04) | –.01 (.04) | .01 (.03) |
|  | CE × Parking | –.03 (.04) | .05 (.03) | .02 (.03) | **.06 (.03)** |
|  | CE × Recreation | .03 (.05) | .04 (.03) | .02 (.03) | –.02 (.03) |
|  | CE × Vacant | –.02 (.04) | –.02 (.03) | –.03 (.03) | –.02 (.02) |
| Past Coll. Eff. *d*-Sep. *p*-value | | .13 | .73 | .06 | .43 |
| *R*^2^ | | .20 | .32 | .38 | .31 |
| *N* = 1,641 for all models; Standard errors in parentheses. | | | | | |
| Bolded estimates significant at 95% level; Trigamma *R*^2^. | | | | | |

1. * Published in *Criminology*, volume 60, issue 2, 2022. [↑](#footnote-ref-1)
2. All tables cited in the appendix are provided at the end of this document. [↑](#footnote-ref-2)
3. The equation for two-level reliability in neighborhood $k$ is $\lambda_{k}=\frac{\tau_{\beta}}{\left( \tau_{\beta}+V_{k} \right)},$ where $V_{k}=\sigma^{2}/J_{k}$. [↑](#footnote-ref-3)
